# Review of Recent Bio-Inspired Design and Manufacturing of Whisker Tactile Sensors

**DOI:** 10.3390/s22072705

**Published:** 2022-04-01

**Authors:** Mohamad-Ammar Sayegh, Hammam Daraghma, Samir Mekid, Salem Bashmal

**Affiliations:** 1 Department of Mechanical Engineering, King Fahd University of Petroleum and Minerals, Dhahran 31261, Saudi Arabia; sayegh@kfupm.edu.sa (M.-A.S.); hammam@kfupm.edu.sa (H.D.); bashmal@kfupm.edu.sa (S.B.); 2 Interdisciplinary Research Center for Intelligent Manufacturing and Robotics, King Fahd University of Petroleum and Minerals, Dhahran 31261, Saudi Arabia

**Keywords:** whiskers, tactile sensors, nanocomposite, cellulose whiskers, mystacial vibrissae

## Abstract

Whisker sensors are a class of tactile sensors that have recently attracted attention. Inspired by mammals’ whiskers known as mystacial vibrissae, they have displayed tremendous potential in a variety of applications e.g., robotics, underwater vehicles, minimally invasive surgeries, and leak detection. This paper provides a supplement to the recent tactile sensing techniques’ designs of whiskers that only sense at their base, as well as the materials employed, and manufacturing techniques. The article delves into the technical specifications of these sensors, such as the resolution, measurement range, sensitivity, durability, and recovery time, which determine their performance. The sensors’ sensitivity varies depending on the measured physical quantity; for example, the pressure sensors had an intermediate sensitivity of 58%/Pa and a response time of around 90 ms, whereas the force sensors that function based on piezoelectric effects exhibited good linearity in the measurements with a resolution of 3 µN and sensitivity of 0.1682 mV/µN. Some sensors were used to perform spatial mapping and the identification of the geometry and roughness of objects with a reported resolution of 25 nm. The durability and recovery time showed a wide range of values, with the maximum durability being 10,000 cycles and the shortest recovery time being 5 ms. Furthermore, the paper examines the fabrication of whiskers at the micro- and nanoscales, as well as their contributions to mechanical and thermal behavior. The commonly used manufacturing techniques of 3D printing, PDMS casting, and screen printing were used in addition to several micro and nanofabrication techniques such as photolithography, etching, and chemical vapor deposition. Lastly, the paper discusses the main potential applications of these sensors and potential research gaps in this field. In particular, the operation of whisker sensors under high temperatures or high pressure requires further investigation, as does the design of sensors to explore larger topologies.

## 1. Introduction

**Figure 1:**
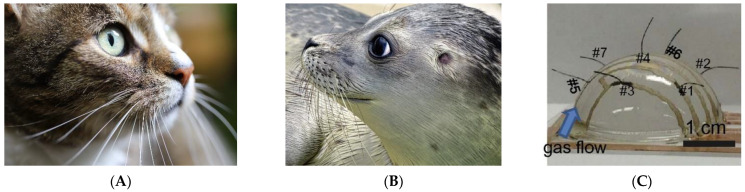
Example of whiskers: (**A**) cat whiskers: (**B**) seal whiskers; (**C**) built micro-whisker sensors [5].

Whiskers or vibrissae are present in almost all mammals and function alone with the skin as tactile sensors to guarantee their safety and health. Mammals such as cats and rats use whiskers (Figure 1A) to identify obstacles and help them navigate through the surrounding environment, especially in dark and narrow places. Other animals such as seals use their whiskers (Figure 1B) to detect water disturbances and to perform seamless hydrodynamic trails by sensing even the weak water movements left behind by passing objects [1]. Furthermore, bats bear whiskers on their wings that provide them with information about airflow [2]. Mimicking such a concept (Figure 1C) represents the aim of building instruments with tactile external or surface sensors that are needed in many engineering applications and that operate in different conditions [3,4].

The external stimuli affecting the structures may lead to intrinsic and/or extrinsic changes, especially in micro-and nanoscale structures [6]. Hence, such whisker sensors have drawn the attention of many researchers, as they seem to possess significant potential in several applications. For example, artificial whiskers can be used in robotics for spatial mapping [7], especially in situations where the operation of optical sensors is difficult due to fog, glare, or reflections [8]. They can also be used to detect and map water or gas flow as well as in leak detection.

An important classification of whisker sensors is whether they operate in active or passive systems. In passive systems, the whiskers have no actuation, and they start sending signals when they pass by an object, such as in simple tactile collision detectors. In active systems, however, the whiskers are actuated by a mechanism that mimics the “whisking” movement of certain animals. Cats and seals, for example, continuously sweep their whiskers back and forth using extrinsic muscles that move the whisker pad [9]. Furthermore, some rodents have intrinsic muscles for each whisker for better control. The study showed that rats also exhibit different whisking behaviors when running, turning, or exploring new environments, which provides them with critical information. Kim and Möller compared active and passive sensing for distance estimation and showed that active sensing can provide significantly more precise measurements [10].

Various types of artificial whiskers have been proposed. Initial designs depended mainly on relatively bulky torque and force sensors [5]. However, with the recent development of materials and nanotechnologies, more practical and promising concepts are emerging such as electronic whiskers (e-whiskers), offering light and compact structures that can be integrated with user interface systems and achieve high sensitivity measurements. E-whisker sensors also present a new class of sensors in addition to nervous materials, but they do not require direct contact with the sensitive material. In addition, the advancement in wireless sensor networking technologies facilitates further progress in sensing techniques where wireless technology is used to perform the sensing in proper and intelligent methods on both macro- and microscales [11,12,13,14,15,16,17].

In this paper, the recent designs of these sensors are reviewed. The challenges associated with these sensors are discussed, such as the selection of host and sensitive materials, sensitivity, durability, and manufacturing. Accordingly, the paper is grouped according to different aspects into several subsections including the direction of sensitivity, materials of both the substrate and the sensing elements, the manufacturing processes used to fabricate the whiskers, and the functionality of the fabricated whiskers in terms of their sensitivity, recovery time, and durability. Moreover, the article presents a couple of research papers that investigated the fabrication of microscale and nanoscale whiskers and the related characterization in terms of their mechanical and thermal behavior for different applications. Lastly, the potential applications and research gaps, followed by a conclusion of whisker design, are presented. The article evaluates the above-mentioned sections within the purview of designing and assessing the performance of the whiskers.

### 1.1. Growth of Tactile Sensors

**Figure 2:**
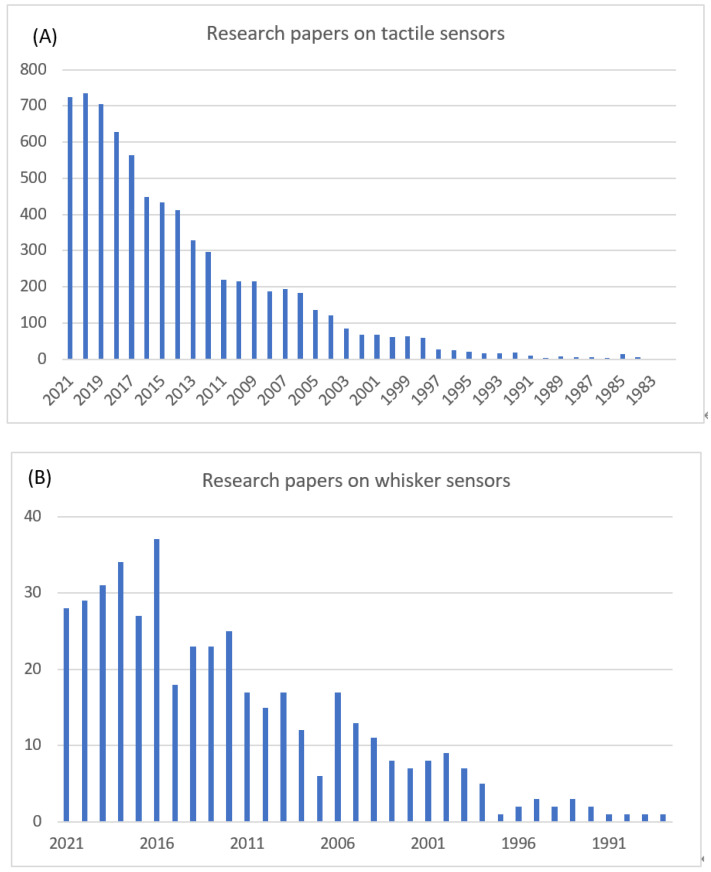
Journal papers per year: (**A**) tactile sensors; (**B**) whisker sensors.

In general, tactile sensors are integrated in various applications to gather information, e.g., force, pressure, temperature, and humidity. There is a growing interest in the development of tactile sensors, which has resulted in a substantial body of research in recent years. Figure 2A depicts the number of articles published each year on tactile sensors over the past three decades [18]. The increase in published papers demonstrates the potential of tactile sensors for a wide range of applications. For example, around 20% of the published articles fall under the “robotics” category. Moreover, a large portion of recent publications focused on exploring the use of new materials, such as nanotechnology, or emerging applications, such as wearable sensors.

Several review studies on tactile sensors have been published to date. The review paper written by Lee and Nicholls thoroughly illustrated the taxonomy and future directions of research [19]. In the past few years, several focused review papers on tactile sensors tackled separate issues in terms of sensing mechanisms, fabrications, and applications. Chi et al. compared the sensing mechanisms, advantages, and disadvantages of recently developed tactile sensors [20]. Liu et al. and Kumar et al. focused on sensors fabricated using 3D printing [21,22]. Other examples of recent review papers evaluated robotics applications [23], graphene-based sensors [24,25], micro- and nano-processing [26], and MEMS-based flow sensors [27].

As illustrated in Figure 2B, research on whisker-like sensors began to evolve in the late 1990s. The data presented in the figure represent the number of journal articles published each year containing the words “whisker” and “sensor” in the title, keywords, or abstract. A comparison of the two figures indicates that, while whisker sensors received increasing attention in the past two decades, the number of research articles still represents a small proportion of the overall papers on tactile sensors. Moreover, although robotics represent one of the application areas for whiskers, only 8% of the published journal papers discussed the use of whisker sensors in robotics. Most of the papers fell into the categories of material science and electronics.

Since there are a wide variety of applications for tactile sensors, there are many key parameters that need to be evaluated. Tactile sensors can be evaluated based on their sensitivity, response time, repeatability, and stability. Inspired by the human body or other creatures, many artificial tactile sensors mimic those found in living creatures. Considering the human skin perception as a reference, the sensor should have the ability to sense forces between 0.01 and 10 N with a response time of about 1 ms [20].

Researchers used principles to transduce measured physical quantities into electronic signals. The commonly used transduction mechanisms are piezoresistive, capacitive, and piezoelectric [21,24,26]. Other tactile sensors used methods such as triboelectric and optical. The piezoresistive effect is one of the most commonly used mechanisms in tactile sensors due to its low cost, wide range of detection, and simple structure. Recent research work focused on the use of newly developed nanomaterials to construct a piezoresistive sensor that is robust and flexible. The use of conductive fillers such as graphene nanowires or carbon nanotubes embedded in an insulated polymer showed promising results [28]. Metallic fillers suffer from coherent incompatibility issues, limiting their application to flexible electronics [20]. Piezoelectric-based sensors have high sensitivity and a fast response. Moreover, they can be self-powered due to their ability to harvest energy. However, piezoelectric sensors are unable to measure static force due to the rapid dissipation of charges [20]. A few sensors rely on the magnetic effect resulting from the movement at the base of the whisker, but they tend to have a relatively larger size [2,29,30,31].

The notable capabilities of tactile sensors have enabled their successful applications in many fields, such as robotics, minimally invasive surgery, human activity monitoring, and Internet of Things. In robotics, tactile sensing is an essential technique to improve robotic manipulation. A sensory surface is mounted on the end-effector to provide any type of information through physical contact with the object. The collected data (usually normal and shear force) are analyzed to define the stable gripping and coordination [19,23]. Piezoelectric micro-whiskers were also fabricated to act as fluid flow sensors [27].

Wide selections of novel materials are promising candidates for tactile sensors following the advance of research in this area, especially soft materials for flexible and wearable sensors. Extensive research work has been carried out in the last few years regarding the characterization of the elastic and electrical properties of sensor materials. Nanomaterials, such as nanowires, nanotubes, and graphene, have attracted the attention of many research groups due to their superior electrical conductivity and mechanical strength [24,25,32]. However, one of the obstacles is the degradation of the mechanical properties at the macroscopic level.

Toward the development of a functional sensor, the three main components are the active material, substrate, and electrode. While the sensing element is the most important layer in the sensor, there is a need for a substrate and electrodes that are compatible with the active layer. Fabrication of a functional sensor with high sensitivity and reliability requires novel techniques. Therefore, several studies investigated various manufacturing methods. The 3D printing technologies are among the powerful tools used in tactile sensor fabrication. Liu et al. reviewed the research progress on 3D printing techniques and the recent advancement of fabrication several tactile sensors using 3D printing [21]. Recently, Niu et al. reviewed various template methods used for the preparation of microsensor arrays [26].

### 1.2. Bio-Whisker Fundamentals

Bio-whiskers were reviewed in various studies, including their related sensing designs. Land and aquatic mammals have whisker-like hairs raised from follicles that are densely packed with nerve endings. Any contact of an object by the whisker is converted into stresses in the follicle. Mechanoreceptors in sensory neurons transduce these stresses into neural signals, which are interpreted by the central nervous system to learn about the structure of the surrounding world.

Across species, whiskers exhibit diverse shapes, which reflect the behaviors and ecological niches of their owners. For example, the whiskers of harbor seals have elliptical cross-sections with an undulating diameter (Figure 6B). This structure enhances the seal’s ability to analyze fine-scale water currents associated with prey movements. The facial whiskers of rodents, called macrovibrissae, have conical shapes with a base of about 100 µm in diameter close to the follicle and about 20 mm length. This conical shape allows rodents to measure object distance using a single whisker, explore rough textures, and prevent the whiskers from getting caught in tight spaces. This graded size defines the bending and locates the touched object with respect to the whisker [9]. Cylindrical shapes as shown in Figure 6C may not have the same characteristics and become stuck in tight spaces. The bending stiffness of a whisker is proportional to the fourth power of the whisker diameter. It is defined as the bending moment required to produce a given change in whisker curvature. Hence, the bending stiffness decreases by five orders of magnitude from the base to the tip, reflecting the decreasing diameter of the conical whisker. In this complex system, rodents employ vector math to locate objects effectively. The whiskers are arranged in a grid on the face of rodents with different lengths at different positions with the possibility to regrow. According to [9], tactile information from each whisker is mapped to topographically arranged groups of neurons throughout the brainstem, thalamus, and somatosensory cortex. A large area of the rodent neocortex, called the barrel cortex because of its distinctive cytoarchitecture, is devoted to processing tactile information from the whiskers, which highlights the importance of whisker-based touch as a sensory modality for these animals.

#### 1.2.1. Motion of the Bio-Whiskers

The motion of whiskers in animals, e.g., cats and seals, is composed of head motion and extrinsic muscles that move the entire whisker pad forward and backward with respect to the head. Others have a specialized system of intrinsic muscles, one per whisker, for fine-scale control of whisker movements. Both muscles allow whiskers to move primarily in an arc in the rostral–caudal direction [9]. This allows the fine-scale positioning of the entire group of whiskers and individual whiskers during tactile exploration, such as digits on the human hand. High-level control is secured by the motor cortex accessed by motor neurons driving the whiskers.

The whiskers can move at a fast rate of around 10,000 degrees per second, revealing a significant and complicated processing network inside to identify shape and position. Furthermore, touch-induced deformation of whisker morphology offers nearly comprehensive information on the forces in the sensory follicles.

The rodents move their whiskers forward (protraction) and regularly regulate whisker position around this protracted position at a well-defined frequency (15–20 Hz mice, 5–10 Hz rats) over dozens of cycles when in exploratory mode. Rhythmic whisking is linked to speed: the quicker rodents run, the faster they whisk and the more their whiskers protract.

When the surrounding environment is known, the rodents gain confidence, which is demonstrated by a reduction in whisking amplitude. In the opposite case, whisking is more often used to perceive surroundings, indicating strong neural control. Because whiskers and their interactions with objects can be observed continuously throughout lengthy behavioral epochs, head fixation is also valuable in the research of tactile activities.

When running on a spherical treadmill, head-fixed mice whisk naturally. Whisking measurements in head-fixed mice were integrated with fine-scale locomotion and smelling data, as well as recordings and manipulations of brain activity. These studies have shown that rhythmic whisking is phase-locked to stepping, sniffing, and even ultrasonic vocalizations, most likely via neural circuits in the brainstem; this synchronization could serve as a shared time base for neural calculations.

#### 1.2.2. Bio-Whiskers Interaction with Objects

Whisking is modulated by touch on many temporal scales. Rats change the direction of their whiskers to prolong protraction and press their whiskers deeper into an item [9]. The whisking is controlled around the item, enabling the integration of data from several touches to increase decision-making precision. Rodents can identify the shape of objects and monitor forces applied to their whiskers through tactile activity. Furthermore, they can discriminate texture roughness.

Many of the concepts discussed later in this paper are interestingly related to the bio-whisker description seen here.

### 1.3. Growth of Bioinspired Whisker Sensors

Designing new whiskers was largely inspired by the above-discussed bio-whiskers. Whiskers are designed tactile sensors that can be used to sense nearby objects and provide spatial information. Analogous to cat whiskers, they provide fast and reliable information about force, pressure, and distance. They can supplement or substitute vision-based sensors, especially for close or out-of-sight objects. Furthermore, they might provide cheaper and replaceable alternatives for other sensors. While there is a significant focus on reviewing the research progress in other tactile sensing technologies, there is a need for a detailed literature review on whisker-based sensors to explore their applications and capability.

Whiskers are exteroceptive sensory organs in felines and other mammals that allow them to navigate through narrow spaces and explore close objects. Both whiskers and antennae are hair-like structures that fall into the category of extrinsic tactile sensors. They provide precise information about the interaction properties of the surrounding objects. They consist of a highly flexible and taper beam. However, while these structures appear similar, they are fundamentally different. Whiskers are more common in mammals, while antennae are found in arthropods. The basic function of whiskers is to feel the nearby movements, either by direct contact or through sensing the movement of the air or water. Antennae have a wider range of functions and more complicated structures that include muscled sections and sensory cells within the structure.

Generally, whiskers are long thick hair without any internal innervation. The bio-inspired whisker-like sensors consist of a single or a network of protruding elements mounted on an active sensing element. The excitations are not applied directly to the sensing elements. The motion of the whisker due to an external disturbance triggers the sensing element, and the signal can be acquired and interpreted. This an important feature in distinguishing the whisker-like sensor from other tactile sensors where the disturbance is applied directly to the sensing element. There are some studies that used different terms (e.g., “stretchable strain sensors”), but the developed sensor possessed similar characteristics and is relevant to the literature on whisker-like sensors. As a principle, all whiskers are flexible springs that have a sensing mechanism at the roots only.

**Figure 3:**
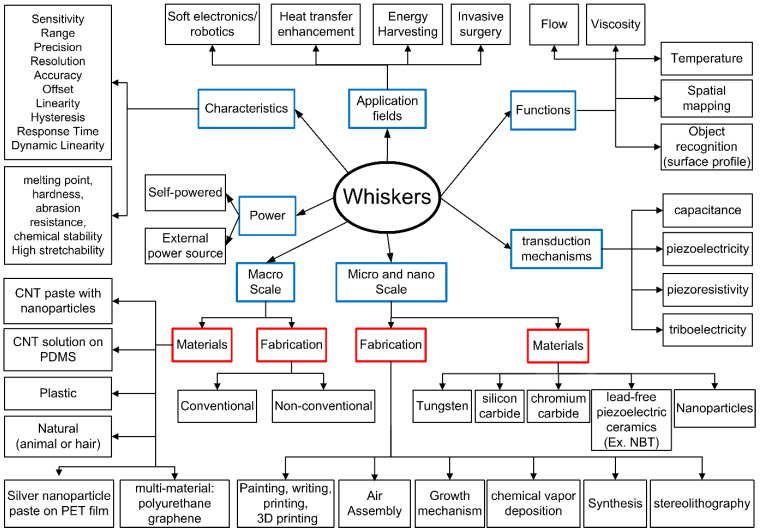
A taxonomy for whisker-based sensors.

Figure 3 illustrates the taxonomy relevant to whisker-like sensors. The focus areas and keywords used in recent research are highlighted and discussed briefly. The keywords are grouped into categories to illustrate the topics of interest and future opportunities. The functions, scale and characteristics are in blue boxes partially leading to related fabrication and materials in red boxes.

Early research on whisker-like sensors used natural (animal or human hair) or traditional materials (plastic, metal) to build the sensor element. The vast majority of recent research papers on the development of whisker-like sensors explored the use of novel materials with superior properties.

In their work, Amoli et al. reviewed the recent biomimetics in tactile sensors with a discussion about whisker sensors using smart design and materials (e-whiskers) [33]. They included recently developed artificial sensory systems with bioinspired functional features, which enable close emulation of the functional aspects of biological systems, such as biological ion channels, skin mechanoreceptors, and sensory neurons, for the fabrication of advanced human–machine interfaces. The review discussed smart materials and devices mimicking human skin. In addition, Tripathy et al. discussed recent sensing technologies inspired by nature including whisker-like sensors [34]. These discussions generally focused on the use of the emerging technologies to build efficient designs. In particular, the recent advancement in nanotechnology inspired several research groups to explore their sensing ability in building whisker-like sensors. Carbon nanotubes, nanoparticle additives, and graphene are among the materials used to enhance the sensing ability of the active element in the sensor. Our discussion focuses mainly on reviewing whisker sensors utilizing these technologies with further elaboration.

Nanomaterials are used in macro sensors to enhance their functionality and performance. The whisker can be coated with a nanoparticle paste or solution prepared by mixing nanoparticles during the fabrication phase. However, the dimensions of the sensor are in the macroscale range. There were several studies on whiskers performed at the nanoscale level. However, the term whisker was used to refer to the shape of the structure with a high length-to-diameter ratio at the nanoscale level. The functionality of the sensor was not discussed. The scope of these research papers involved the enhanced properties or development of a cost-effective fabrication method and not in the building of a sensor at the nanoscale level. Vertically aligned carbon nanotube, chromium carbide, or NBT whiskers represent a few examples from the recent literature.

There were, however, few attempts at the microscale level to develop networks of whisker-like sensors. It is important to note that those sensors may not be referred to as whiskers but as nanowires, nanorods, nanopillars, nanotubes, and nanoribbons [35,36]. In principle, they also consist of a flexible rod mounted on a sensing element with the purpose of responding to any external stimuli.

The development of artificial whiskers with high performance and superior properties, requires new techniques in material selection and fabrication methods. Moreover, the need for miniaturized and flexible sensors requires the use of novel fabrication techniques. Therefore, recent research involved the fabrication of miniaturized sensors using various innovative fabrication methods. For example, growth mechanism, chemical vapor deposition, synthesis, painting, stereolithography, writing, and printing are among some keywords that were frequently used in the recent literature. While some of the described methods are traditional, the focus was usually toward fabrication on the micro- or nanoscale level. Among novel fabrication methods, 3D printing received special attention from researchers in the past decade, being used to fabricate single- or multi-material whiskers with high aspect ratios. It was also proven to be a powerful rapid prototyping tool to build customized low-cost sensor models.

Whisker-like sensors convert the measured physical quantitates into electrical signals. The commonly used approaches are piezoresistive, capacitive, and piezoelectric. It can be observed that the vast majority of whisker-like sensors measure the resistance change of the active material due to the movement of the whisker. Typical strain gauges were used due to their cost-effectiveness and high sensitivity. However, several works employed novel materials to replace the conventional strain gauges. Piezoelectric-based sensors offer the opportunity to develop a self-powered sensor that does not need an external power source.

Whiskers typically measure the force and pressure that cause a deflection. Following the same principle, they can be used to measure the flow, viscosity, and strain. They have been used as temperature sensors by observing the resistance change due to variation in the temperature. Spatial mapping and object recognition are some of the direct applications of whiskers, and they were among the first applications inspired by bio-whiskers. Applications such as heat transfer enhancement, energy harvesting, and invasive surgery are also potential applications where whiskers show promise.

The sensor performance is demonstrated by examining its characteristics. There are several common parameters used as performance measures in the evaluation of sensors. They are grouped together according to the taxonomy, and they have to be evaluated to illustrate the capability of the sensor.

The other groups of parameters in the taxonomy are specific to certain materials or applications and may not apply to all types of sensors. For example, high stretchability is essential in whiskers since they require high strain resolution.

## 2. Tactile Sensing Techniques and Related Sensitivity

### 2.1. Tactile Sensing at a Macroscale

Whiskers mainly translate the information of objects to be sensed when they are directly in contact. Due to the direct contact, the mechanical properties, size, and physical shape of whiskers are essential characteristics [37]. The whiskers of mammals vary in length, shape, thickness, and stiffness [38]; these variations depend on the size of the mammal and the function of its whiskers. Therefore, mimicking this concept using artificial whiskers on the macroscale should consider the mechanical whisker properties and especially actuation mechanisms used to suitably achieve the sensation [39,40]. Furthermore, as introduced previously, passive and active whisking constitute a fundamental concept in mimicking artificial whiskers.

**Figure 4:**
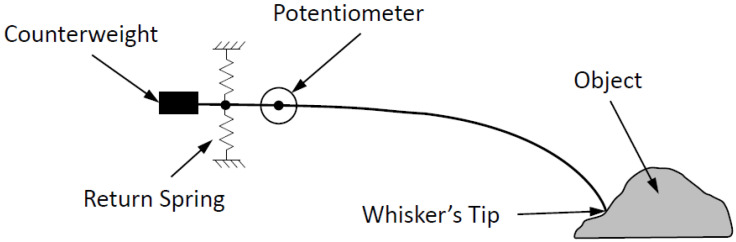
Example of hinged whiskers [42].

Before proceeding with very precise models, whiskers were modeled as elastic beams with hinges [41] referred to as hinged whiskers, shown in Figure 4 [42] to illustrate the passive whisker system. The deflection in the hinged whiskers is measured by the rotation in the potentiometer due to the tip directly touching the sensed object, while the springs play a role in returning the whiskers to their initial position.

**Figure 5:**
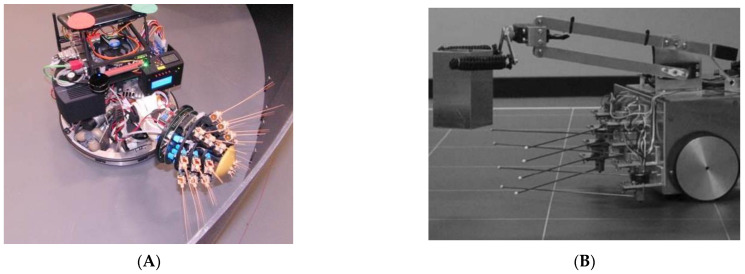
Examples of whiskered robots: (**A**) Shrewbot [43]; (**B**) Whiskerbot [44].

As an advanced step (i.e., active whisker system), mechanical mechanisms were replaced with electronic ones (e.g., robots [40]), where the built artificial whiskers were installed on robots. For example, whiskered robots use macroscale whiskers through a variety of actuation mechanisms to mimic the active whisker systems in animals. The “Shrewbot” whiskered robot [43] shown in Figure 5A has 18 artificial whiskers that are mounted on a mobile robot. “Whiskerbot” shown in Figure 5B uses different actuation mechanisms; it has rigid steel wires that are able to move/rotate and consequently characterize the shape of the contacted objects [44].

### 2.2. Tactile Sensing at a Micro-/Nanoscale

Usually micro- and nanoscale materials exhibit superior mechanical, thermal, and electromagnetic properties that vary from those of macroscale materials [45]. Thus, researchers are interested in involving such materials in different applications to enhance the material properties.

Wang et al. [46,47] synthesized a chromium carbide micro-whisker using chromic oxide, black carbon, halide salts, and nickel. The obtained micro-whiskers were characterized, showing thermal stability in terms of oxidation up to 1000 °C. They also added that the Cr3C2 micro-whiskers played the main role in improving the bonding strength of heat-resistant adhesive by 16–28% in the temperature range of 500 °C to 1500 °C. The synthesized micro-sized whiskers were also used to enhance the heat transfer of fluid-laden whiskers. Zhang et al. reported that fluid flow containing silicon carbide (SiC) whiskers showed a significant improvement in the heat transfer coefficient compared to the base fluid [48]. For 0.16 wt.% of SiC-laden water, the fabricated SiC whiskers increased the heat transfer in the range of 37–43 °C. These results can enable further investigations to optimize the contribution of the shape and the concentration of whiskers to achieve greater improvement of heat transfer fluid applications.

For the purpose of studying the mechanical behavior of tungsten (W) whiskers, Liu et al. used the CVD process to prepare hexagonal cross-sectioned W-whiskers tested under nanoindentation [49]. Jiang et al. synthesized lead-free Na_0_._5_Bi_0_._5_TiO_3_ (NBT) whiskers and characterized their piezoelectric properties [50]. The authors reported the possibility of using NBT whiskers (lead-free whiskers) as an ideal component in energy-harvesting applications and microelectromechanical systems. The molten salt synthesis (MSS) method was used to fabricate the NBT whiskers from layered tunnel-structured Na_2_Ti_6_O_13_ (NT).

### 2.3. Sensitivity

The sensitivity of the sensor describes its ability to detect small mechanical stimuli, e.g., forces or pressure, that can be described as the change in electrical measurements, i.e., the change in output for a change in input. In this section, the sensitivity of the whisker sensors is discussed. Due to the electrical conductivity property of the whiskers, their detection ability is typically measured according to the resistance change across the strain gauge; thus, the sensitivity or accuracy can be described as the change in resistance per unit force (ΔR/R; N), unit pressure (ΔR/R; Pa), or unit speed (ΔR/R; m/s) in terms of measuring the speed of fluid flow. These quantities are called gauge factors. However, others reported the sensitivity in terms of deflection (i.e., ΔR/R; mm), which renders comparisons of the sensitivity difficult, especially when the required force to cause the deflection is unknown. Therefore, the discussion here is grouped according to the considered measurements.

Pressure-based whisker sensors have been used for monitoring fluid flow in various applications (e.g., gas flow and underwater flow). For instance, Takei et al. tested the sensor sensitivity under airflow, revealing low sensitivity (approximately 8%/Pa or 13.3%/m/s) [5]. The sensor designed by Liu et al. showed a slight improvement when tested under a nitrogen flow, revealing a 20% change in resistance in the range of 0.3–1.5 m/s [51]. The previous work by Wu et al. which relied on signals induced by magnetic flux was capable of detecting airflow as low as 1.2 m/s [29]. Furthermore, a high-pressure sensitivity of 58%/Pa was reported with a response time of around 90 ms [52]. Liu et al. tested a microscale whisker sensor for underwater applications which could detect a flow of 0.1 m/s; it was capable of distinguishing between laminar and turbulent flow [51]. The sensor designed by Kottapalli et al. could detect flow as low as 193 μm/s [1]. Moreover, Zeb et al. reported high sensitivity for water vortex detection with a resistance change of 1180%, but the intensity of the vortex was unclear [53].

In contrast, the sensitivity of other sensors was measured in terms of the resistance change per force applied, such as [54], where a sensitivity of 0.313/N (31.3%/N) and a resolution of 0.002 N were achieved. Wakabayashi et al. measured a maximum sensitivity of 0.56/gF (57.1%/N) [55]. Furthermore, a sensor depending on the piezoelectric effect could detect forces with a resolution of 3 µN and a sensitivity of 0.1682 mV/µN with good linearity [56].

For spatial mapping, several sensors were tested to identify the geometry or roughness of objects of different sizes. In a study by Reeder et al., an array of whiskers was used to scan a fingerprint mold and detect ridges as small as 25 µm; it was also able to scan the grains of a leather texture [57]. In addition, it was used to differentiate surface roughness ranging between 42 and 657 nm. Similarly, sensors were designed for use in spatial mapping and could detect steps with a resolution (in terms of height difference) of 50 µm [58] and 400 µm [52]. The pencil-drawn paper whisker had a relatively lower resolution of around 2 mm. Precision positioning applications could be of interest.

### 2.4. Direction of Sensitivity

**Figure 6:**
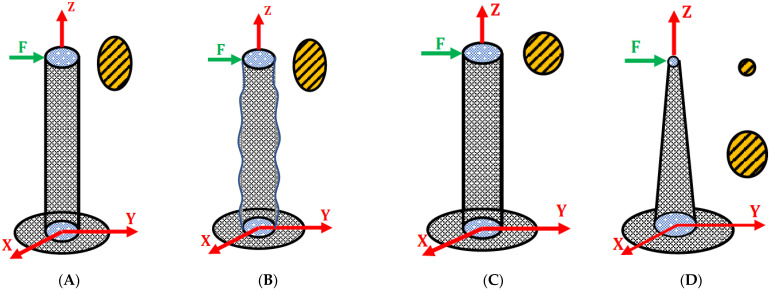
Schematic of elliptical cross-section (**A**) moving in the direction of the force, and circular cross-section (**C**) interacting with the applied force (i.e., deviation depends on the applied force direction). Both can feature a wavy surface of the whisker (**B**) or a conical shape (**D**).

There are various whisker designs with different abilities to distinguish the direction of contact, which is an important feature for some applications. The direction of detection can be along one axis or more depending on the way the sensitive material is patterned, and the geometrical shape of the whiskers may constrain the deformation to a certain direction. For example, the elliptical cross-section in Figure 6A supports the advantage of bending in one direction compared to the circular one in Figure 6B under different directions of the load.

#### 2.4.1. Detection along One Axis (Up or Down)

**Figure 7:**
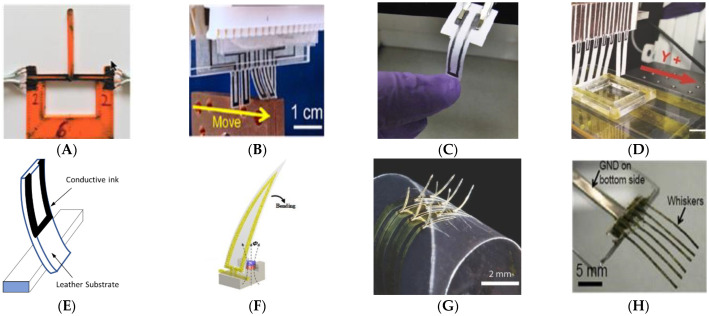
Whiskers with detection along one axis (up or down) using different substrate material: (**A**) Ninjaflex substrate [62], (**B**) plastic substrate [52], (**C**,**D**) network of conductive graphite particles in a thin layer [63,64], (**E**) leather substrate [58], (**F**) Ecoflex substrate [29], (**G**) polyimide film [57], and (**H**) PDMS layer [5].

This type of whisker can only distinguish a force perpendicular to the whisker that causes it to bend up or down, as shown in Figure 7. The design is relatively simple and typically consists of a substrate, such as Ninjaflex (Figure 7A), plastic (Figure 7B), a network of conductive graphite particles in a thin layer (Figure 7C,D), leather (Figure 7E), Ecoflex (Figure 7F), polyimide film (Figure 7G), or PDMS layer (Figure 7H), with patterned sensitive material on one side. As the whisker bends, the material is subjected to compression or tension, which increases or decreases the resistance. In other designs, different mechanisms were used for sensitivity, such as that proposed by Delamare et al. [59] which relied on capacitive change for detection, or that proposed by Ju and Ling [60,61] which used a piezoelectric bimorph with sensing and actuating abilities. However, both cases were still confined to force detection along one axis.

#### 2.4.2. Detection along Two or Three Axes

**Figure 8:**
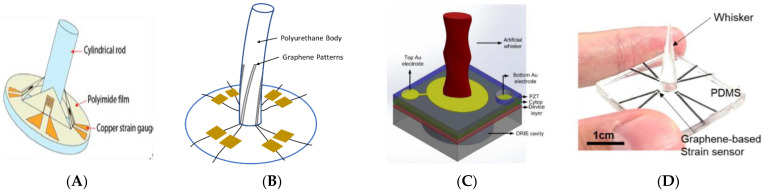

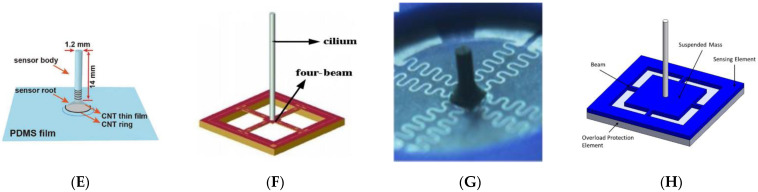
Whiskers with detection along two or more axes: (**A**) graphene filament connected to cylindrical whisker [54], (**B**) 3D-printed cylindrical polyurethane [53], (**C**) (SOI) wafer whisker attached to PZT base [1], (**D**–**G**) Force detection whiskers with PDMS substrate [29,51,65], and (**H**) quartz fiber body attached to SOI wafer [56].

The sensors shown in Figure 8 are examples of two-axis whisker sensors. The whisker body is placed in the center with the strain-sensitive material placed around it, which allows the whisker to detect the applied forces in 2D, confirming the concept of using a circular cross-section (Figure 6B). Moreover, simultaneously monitoring the resistance change in all the strain gauges can be used to identify the direction of the force around the whisker, but with a reduced accuracy at certain angles, as demonstrated by [51,54,55]. Previous research introduced the concept of detection along two and three directions using different substrate materials at the base connected to a set of sensorial materials such as graphene on the cylindrical whisker, as shown in Figure 8A. Another example is the 3D-printed cylindrical polyurethane whisker used for vortex detection applications (Figure 8B). In the same context, the silicon-on-insulator (SOI) wafer whisker attached to PZT base in Figure 8C was used to detect water flow. However, a whisker with a quartz fiber body was also attached to the SOI wafer to detect mechanical behavior in minimally invasive surgeries (MIS), as shown in Figure 8H. Figure 8D–G show that the complexity of the out-of-plane flexible whisker is increased to satisfy the requirements of various applications using PDMS substrate, which mainly work on the basis of force detection along two and three directions [65].

**Figure 9:**
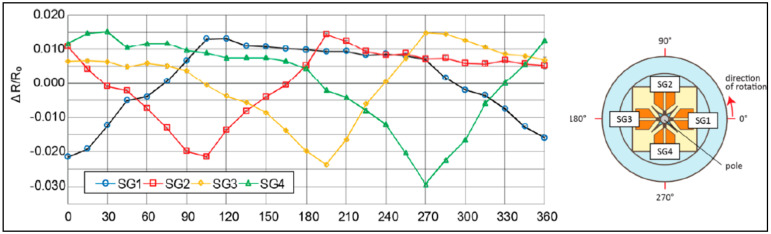
Resistance change of strain gauges due to force in different directions [54].

The graph in Figure 9, shows the change in resistance of four strain gauges equally patterned around the whisker as the force direction changes from 0° to 90°. Similar results were provided by [51,65]. In the study by Kottapalli et al., the whisker had an elliptical cross-section that caused different responses when the direction of water vibration changed, giving it directional sensitivity [1]. However, it is not clear how the whisker will detect the direction when the intensity of the vibration is variable. Some sensors can also detect forces along a third axis along the whisker (coaxial force), as described in [51,56,66].

## 3. Core Materials in Tactile Sensors

The most important aspect in the design of whisker sensors is the type of material used in both the substrate and the strain gauges. The sensitivity of the whisker and its robustness are directly linked to the type of the material used. The flexibility that the sensor’s body should have, which enables it to sense any external influence on its smallness, should be considered during material selection; to achieve this purpose, PDMS due to its relatively lower density of 965 kg/m³ is commonly used as a substrate. In addition, electrical conductivity is necessary to perform electrical measurements, which help to quantify the strain as electrical resistance; thus, different materials such as carbon nanoparticles, carbon nanotubes, and silver nanotubes are frequently used for sensing strain due to their good electrical conductivity. The diversity of applications has led to the use of other materials such as gold, chromium, or lead as electrical-based strain gauges. Moreover, different host materials have been used such as 3D-printed PLA, leather, or paper to host these sensing elements. This section briefly discusses this aspect.

Takei et al. used a PDMS layer with a thickness of 250 μm as a substrate painted on a thin film (i.e., ~2 μm carbon nanotube paste (CNT) and silver nanoparticles (AgNPs)), as shown in Figure 7H [5]. The strain sensitivity of the composite was highly increased by adding AgNPs to CNT; however, its resistivity was decreased. The authors proved that changing the content ratio of the CNT and AgNP composition could lead to a change in its characteristics. The authors used 5 wt.% and 30 wt.% AgNP composites in the top and bottom electrodes of the whisker, respectively, which made the former more resistive, thus affecting the outputs of the sensor. However, the usage of AgNPs alone without nanotubes resulted in irreversible degradation under strain, which is impractical for cyclic loading. On the other hand, patterned silver nanowires (AgNWs) were embedded within a PDMS to create a film which was attached to a magneto whisker; accordingly, this was used for tactile and flow detection [66].

Moreover, PDMS was employed as a substrate to build a whisker through exposing polyimide films to a laser, as shown in Figure 8D; the laser was used to transfer the graphene to the PDMS as a nanocarbon, which was used as a strain sensor (laser-induced nanocarbon) [55]. In the same context, Liu et al. used single-walled carbon nanotubes (SWCNTs) as a sensitive material due to their high stretchability [51]. The whisker body consisting of a PDMS film with PLA was covered by a film of SWCNTs, as shown in Figure 8E. To bond the copper wires with the SWCNTs, a liquid metal (gallium–indium eutectic) was used as the conductive adhesive, which largely improved the stability of the structure.

Other types of polymer substrates were used in previous studies; for instance, Zeb et al. used a 3D-printed polyurethane shaped as a cylinder to build a whisker for vortex detection applications, as shown in Figure 8B [53]. The authors attached four patterned graphene filaments around the cylinder using copper tapes as connectors. As shown in Figure 8A, a similar design was proposed where a sensor was built using a stainless-steel pole, mimicking the anatomy of a tooth [54]. The pole was attached to four surrounding strain gauges composed of a polyimide film with chromium and copper vapor-deposited layers, as shown in Figure 7G. A shape memory polymer (SMP) was used by Reeder et al. for the whisker’s body, which could be extended or retracted by heating or cooling [57]. Sensitive gold strain gauges were patterned on top of the whiskers. The temperature softened the SMP substrate and reduced its modulus of elasticity, which significantly decreased the minimum bending radius by 85%, making the whiskers more flexible.

Ninjaflex is a flexible nonconductive material fabricated through the 3D printing process, with the capability to be used as a substrate for whisker applications where flexibility is needed. Eijking et al. used such a material to build a whisker, as shown in Figure 7A, while the strain gauge was printed using a filament made of a conductive rubber-like material (PI-ETPU 95–250 carbon black) [62]. Similarly, Ecoflex substrate was used to build a sensor inspired by spider hair for wind-sensing applications, as shown in Figure 7F [29]. A polyethylene terephthalate (PET) film with patterned silver nanoparticles (SNPs) was fixed to an Ecoflex substrate with an Nd_2_Fe_14_B magnetic cube, which caused magnetic flux to pass through the SNP pattern and generate a current.

With a plastic substrate, a CNT–AgNP composite (Figure 7B) was also used to build a multifunctional artificial electronic whisker in [52]. The authors reported a high sensitivity to resistance change for the composites with a 45 wt.% concentration of silver nanoparticles. The design also incorporated a temperature sensor using a (PEDOT:PSS)–CNT composite film (poly(3,4-ethylene dioxythiophene)–poly(styrene sulfonate)–CNT), and it was found that the resistance decreased as the temperature increased with a sensitivity of ~0.63%/°C.

The natural porosity of leather enables its absorption properties, while the fluidity of conductive ink allows it to be absorbed by leather, thus protecting it against external corrosion and, consequently, securing good durability. Xie et al. used leather as a substrate for a durable electronic whisker with conductive ink, as shown in Figure 7E [58]. Carbon black (CB), a carbon nanomaterial, was used due to its ability to permeate into the leather during the printing process. Other materials such as SWCNTs and graphene oxide (GO) were also tested, but they were more suitable for deposition on the leather surface and, therefore, vulnerable to surface damage. Furthermore, SWCNTs displayed poor sensitivity and linearity in bending, while GO showed ultrahigh resistance for such an application. Paper is not used frequently for building whiskers; however, pencil traces on paper were used as strain gauges for whiskers in [63,64]. The pencil traces formed a thin layer of a conductive graphite particle network, as shown in Figure 7C,D. Expansion and contraction of this network due to strain affected the conductivity. Lin et al. found that traces drawn with harder pencils (HB) with a lower content of graphite particles had lower resistance and exhibited greater responses to the resistance change with strain, while trails of softer pencils such as 9B exhibited a lower response. High-aspect-ratio Si60 polymer was used to fabricate a micro-whisker using stereolithography [64]. Kottapalli et al. attached a micro-whisker to a 300 μm thick silicon wafer, which played the role of substrate for the conductive LCP 3908 film consisting of a 25 μm film with 18 μm copper cladding on both sides; the silicon substrate and LCP film were adhered with a 2 μm thick SU8 2002 spin-coated material [67].

Kottapalli et al. attached the fabricated micro-whisker to a piezoelectric base composed of an SOI wafer attached to a Pb(Zr_0_._52_Ti_0_._48_)O_3_ (PZT) plate and two Cr/Au electrodes, as shown in Figure 8C [1]. Ju and Ling also fabricated a micro-whisker transducer (μWT) [61], which consisted of a piezoelectric bimorph (i.e., PZT) with 500 μm width, 500 μm thickness, and 32 mm length, connected to plastic microsphere tip of 340 μm radius via a micro tungsten wire with a 50 μm radius and 3.2 mm length. The μWT was fabricated using special electrical discharge machining in microscale (μEDM). The manufacturing methods generally used to build these whiskers are discussed next.

Before moving on to manufacturing techniques, it is important to highlight the fact that design parameters can vary widely depending on the applications. These can be classified into structural geometric parameters where the shape of the whisker cross-section becomes important, in addition to the length and its shape from root to tip. Other parameters include material properties that can be partly or fully functional (e.g., sensitivity according to material or operation), as well as any added components with structural support or with a specific function (e.g., spring).

## 4. Manufacturing Methods

Since these types of sensors are relatively new, different specific fabrication techniques have been used. However, instead of using the conventional embedding process involving a sensor within the substrate [68,69,70,71], some processes such as PDMS molding and 3D printing are commonly used. In addition, screen printing, photolithography, and lift-off processes were utilized to manufacture parts on a much smaller scale. In some previous studies, simple approaches such as manual painting or drawing could be useful for prototyping. The challenges associated with manufacturing the sensors are generally in integrating the sensitive element with the structure of the whisker while maintaining good durability under cyclic loading, which are usually encountered when working with sensitive materials such as carbon nanoparticles and nanotubes. This required many researchers to perform several chemical processes to build a sensor with suitable properties, becoming more challenging when completely embedding the sensitive material within the structure of the sensor, which is favorable for operation of the sensor in harsh conditions. In nano and microscales, the morphologies of the whiskers represent the main challenge in the synthesis process. Different issues have been reported such as entangled, agglomerated, pointed, and non-whiskered shapes that constrain the movement of the whiskers, leading to measurement uncertainty. This section discusses the manufacturing aspect of each reviewed sensor design in further detail.

### 4.1. Normal Scale Manufacturing

As 3D printing is a favorable process in such applications, it was used as the first step to build the mold receiving the constituents of the whisker. A PLA mold was built via a 3D printing process, and PDMS was later spin-coated [51]. Part of the mold was left in PDMS to act as a stiffener, and the substrate was treated with oxygen plasma to render it hydrophilic. Then, an SWCNT solution was dropped onto the substrate; due to the capillary force, the solution covered the base of the whisker and slightly climbed over the sensor body, which also formed small rings on the body and root after evaporation. Similarly, a 3D-printed mold was filled with induced graphene from polyimide exposed to a CO_2_ laser, creating a film with a specific shape used as the sensitive element [55]. Subsequently, a PDMS solution was poured over the graphene and into the mold; thus, the finished sensor had the graphene-sensitive material embedded in the PDMS body. Takei et al. used deep reactive ion etching to create a silicon mold (250 µm deep, 250 µm wide, and 15 mm long) for a whisker substrate made by pouring PDMS in the silicon mold [5]. Afterward, the substrate was treated using oxygen plasma for improved adhesion; a CNT–AgNP composite was painted on, followed by heat treatment.

Customized 3D printing processes are also able to print the whole sensitive material. For instance, Zeb et al. were able to print a sensor using a customized 3D printer with two extruders: one extruder printed the polyurethane whisker’s body, while the other had a conductive graphene filament to print the sensing element [53]. A simpler design was also fully 3D-printed using a flexible nonconductive material called Ninjaflex and flexible carbon black on the top as a strain gauge [62]. Moreover, Delamare et al. used 3D printing with PLA and highlighted the importance of layer orientation in printing [59]. When the layers were stacked vertically, the whiskers could be easily ruptured, whereas printing the layers at an angle improved the robustness of the structure.

The distribution of the filler within the host material represents an important aspect that requires consideration when building the sensitive element. Thus, screen printing was used to pattern these fillers to obtain the proper dispersion. Screen printing was used to pattern a U-shaped CNT–AgNP film onto several substrates including polyethylene terephthalate (PET), polyethylene (PE), and silicone with oxygen plasma treatment, and then the whiskers were cut using a laser cutter [52]. Similarly, screen printing was also used by Wu et al. to pattern silver nanoparticles on a PET film, in addition to the use of a laser cutter to define the final shape; the design had an Ecoflex substrate produced by molding [29]. In the same context, the advanced photolithography process was used to place gold strain gauges on a shape memory polymer (SMP) substrate, in addition to laser cutting to shape the composed layers into whiskers [57]. Heating was then used to extend the whiskers out-of-plane with subsequent cooling to fix them in the desired position.

More precise manufacturing processes have been used to build sensitive materials. For example, stereolithography was used to fabricate the sensor body. For instance, Kottapalli et al. used stereolithography (SLA) to fabricate a sensor with dimensions and geometry mimicking a real seal’s whiskers [1]. The PZT base was made by attaching the PZT plate to the SOI wafer using the top layer, followed by chemical–mechanical polishing (CMP) to achieve the final thickness. Then, two Au/Cr electrodes formed the contact pads covering the PZT plate on both sides. Finally, to reach the lower electrode, an opening was made by wet etching. The lift-off process is an additive manufacturing process used in micro-structuring technology; the process is aimed at creating the structure of the required material on top of a substrate. Ridzuan and Miki performed the lift-off process to construct strain gauges [54]. A photoresistor was spin-coated over a polyimide film after an appropriate adhesive was applied. After baking, a photomask was set on the resulting film, exposed to ultraviolet (UV) light, and baked again. To adhere the film to the wires, chromium (Cr) was vapor-deposited over the film after oxygen plasma treatment, and another layer of copper was vapor-deposited over the Cr layer, which was used for soldering. The sensor body comprised a stainless-steel pole glued to an acrylic base, and it was inserted through a cut into the strain gauge. Micromachining technology was used to manufacture the whisker. Series of thermal oxidation and etching processes were applied to form the base. The whisker body was made of quartz fiber with a sphere at the tip for safety in MIS applications [56].

Nevertheless, relatively simple approaches have been used to produce sensitive elements embedded within the substrate [66]. The fabrication process first involved PDMS spin-coating on a silicon wafer, and then the AgNW solution coating was applied over a masking film several times and left to dry. Then, the masking film was removed, and the wires adhered to the electrodes. Finally, PDMS spin-coating was reapplied, and a magneto whisker was adhered to the middle of the substrate. Xie et al. used conductive ink (i.e., carbon black) dripped onto a cow leather substrate, which was naturally absorbed into the leather and left to dry; this process resulted in a 3D sensitive element with high and durable sensing properties [58]. Moreover, sensing elements were generated using graphite pencils drawn by hand on paper substrate [63,64].

### 4.2. Micro- and Nanoscale Manufacturing Processes

In micro- and nanoscales, the manufacturing process takes different directions by changing the constituents of the based material. Micro- and nanoscale whiskers are mainly synthesized through growth mechanisms in different environments using optimum parameters. In several studies, the growth and strengthening of whiskers were investigated during the growth mechanism to achieve certain outcomes.

A chromium carbide micro-whisker was synthesized by Wang et al. to build a fundamental understanding of the micro-whiskers growing in preferred directions under severe conditions [46,47]. The authors used a mixture of chromic oxide, black carbon, halide salts, and nickel. The mixture with determined quantities of these constituents was calcined at 1300 °C for 2 h. The liquid-phase catalysis-assisted carbothermal reduction method was used to produce the Cr3C2 micro-whiskers, created through the S-L-S reaction process. Zhang et al. described the production method of the SiC whiskers as follows: a mixture of carbon fibers as a source of carbon and white ashes from rice hulls as a source of silicon was calcined in argon, followed by successive processes of purification, which resulted in SiC whiskers 200–400 nm in diameter and tens of microns in length [48].

Tungsten trioxide (WO_3_) was used to generate volatile tungsten oxide hydrate (WO_2_(OH)_2_) via a reaction with water vapor. A reduction process was carried out for the (WO_2_(OH)_2_) to produce the vapor of tungsten which was deposited on single-crystalline Si substrates. The produced tungsten whiskers had a diameter of about 1–2 μm. In the same context, various factors (temperature, holding time, and position of the Si substrates) were studied by Liu et al. to demonstrate their impact on tungsten whiskers grown using the chemical vapor deposition (CVD) method [72].

Baik et al. fabricated diamond nano-whiskers using the air plasma etching process, which can be used in field emitter cathodes and composite materials, as proposed by the authors. This process was achieved in different stages. First, a hot filament CVD process was used to build polycrystalline diamond films of 3–4 μm thickness as the source of the nano-whiskers formed later. Then, molybdenum (Mo) was deposited for less than 60s with a deposition rate of 0.6 nm/s as an etch-resistant mask [73]. Finally, the diamond substrate was etched by RF or DC plasma to form the whiskers. The physical shape of the created whiskers was affected by the etching parameters; the authors fabricated diamond whiskers with a length greater than 2 μm, a diameter of 60 nm, and a density of 50/μm^2^ using optimum parameters.

Using the soldering process, Tian et al. studied the directional growth of Sn whiskers on an Al substrate to investigate the effect of intermetallic induction on the growth of whiskers [74]. An Al substrate with dimensions of 15 mm × 15 mm × 1 mm was pretreated to secure the deoxidization before the soldering process. The soldering process was completed for a 0.2 g Sn–3.5 g Ag solder at 250 °C for 30 s, before cooling to room temperature. The solder was ground, polished, and cleaned in an acetone solution. Finally, the samples were immersed within dimethyl silicone oil at 100 °C for different durations to allow the growth of whiskers, which were later characterized in terms of their growth orientation. The authors stated that the whiskers grew with misorientation lower than 2° with respect to the underneath grain.

Another fabrication technique was used to grow microscale whiskers as tips for atomic force microscopes to fill a gap in mesoscale measurement. Two-photon adsorbed photopolymerization (TPAP) offers a technique to transform three-dimensional computer-aided design data (CAD) into three-dimensional microstructures using the layer-by-layer accumulation of sliced CAD data. This technique uses a nonlinear light source to enable the polymerization of photoreactive chemicals [75].

However, various techniques were proposed to produce the whiskers such as sol–gel template synthesis [76], a soft lithography approach [69] for the fabrication of nanostructures with well-defined dimensions and shapes, molten salt synthesis (MSS) [71], and chemical vapor deposition (CVD), which produced whiskers with a uniform straight shape, good rigidity, and high-density aligned whiskers [72].

## 5. Durability, Cyclic Loading, and Recovery Time

Whisker sensors undergo frequent bending deformation during operation; therefore, they should be robust and capable of withstanding repeatable deformation. This is specifically challenging because the whiskers are typically composed of different materials forming the substrate and the strain gauge.

The sensors are usually tested by placing them under cyclic loading while observing their integrity and observing whether there is any change in the sensitivity. Most sensors described herein were stable. For instance, several authors tested the designed whiskers for 10,000 cycles and confirmed stability [51,55,58]. Similarly, other studies tested the whiskers for relatively few cycles (i.e., 2000 cycles [53,63], and 1600 cycles [57]) without notable degradation. Furthermore, Takei et al. and Harada et al. tested whiskers for 500 and 200 cycles, respectively, with little or no resistance change [5,52].

Another important requirement of these sensors is protection against the surrounding environment since they rely on direct contact with objects or flow to extract information. Many of the reviewed sensors had the sensing element placed over the surface, rendering it vulnerable. Thus, Wakabayashi et al. embedded the sensing material within the PDMS body, which provided protection against scratching [65]. Similarly, Xie et al. embedded the sensing element inside a leather substrate, and the resulting sensor showed superior resistance to washing and abrasion [58].

Recovery time is defined as the time needed for the sensor to return to its initial state after the removal of the external stimuli. For optimum operation of the whisker sensors, it is required to minimize the recovery time to enhance the resistance change readings and reduce their fluctuation. In the work by Hua et al. and Xie et al., the designed sensors with a substrate made of paper and leather displayed recovery times of 76 ms and 40 ms, respectively [58,63], whereas Wakabayashi et al.’s sensor had a relatively high recovery response of 1.8 s, which was attributed to the properties of the PDMS substrate. The authors highlighted the need to develop a polymer material with better properties to address this issue [55]. Takei et al. illustrated that the sensor’s substrate made of PDMS had a recovery time of 100 ms [5]. Moreover, Ridzuan and Miki had a recovery time of 5 ms, but these differences could be due to the differences in the design or the shape of the whisker and the magnitude of the applied load [54].

## 6. Applications

Whisker sensors can be employed in several applications, especially in robotics, since they are inspired by animal whiskers, and most sensors reviewed in this article were designed for this purpose. They can provide important information to help the robot in navigation, recognition of surrounding objects, or detection of airflow. They can also be used by unmanned underwater vehicles (UUVs). These vehicles require light and low-powered sensors to provide feedback about the surrounding environment. Thus, the development a system is expected to collect and perceive data for navigation. Moreover, some sensors can be used in minimally invasive surgeries (MIS) where laparoscopic instruments are inserted in small incisions in the skin. In certain situations, the use of whisker sensors might become an essential requirement or even enable new applications where conventional sensors such as optical or ultrasonic sensors are not useful (e.g., in dark areas and underwater). Robots are still unable to match the capabilities of animals in tactile sensing, and whiskers have an essential function in this area. Whiskers can explore surfaces from a small distance, which can be important when the surface might cause damage. In some nocturnal underground species, whiskers are more important than eyes [39]. In Table 1, some applications are listed with a short description of the functions of such sensors.

**Table 1:** Whisker sensor applications.

Applications	Enabled Functions and Features
Biomimetic tactile	**Collecting tactile information**: Enhancing the extracted tactile sensing feedback data i.e., human fingertip, resulting from the direct external stimuli by measuring the deformation in prosthetic hands which are measured by means of electrical impedance [77]
Tactile sensors for robotics	**Recognize the surrounding objects**: Whiskers are used to determine the three-dimensional (3D) location of the contacted object [78,79]. **Study the surface texture**: Whiskers can be used to show the surface texture accurately by analyzing the obtained data from the whisking against different surfaces [58,80]. (Figure 10) **Detecting gases’ flow**: A highly sensitive whisker can be used to detect a real-time gas flow in two and three dimensions [5,29]. **Obstacle recognition**: Whiskers are used to sense the environmental obstacles by detecting the surrounding environment in different directions [65]. **Tactile**: Sense motion of heart valve due to its flexibility [81].
Navigation in dark or for blinds	This can be useful for autonomous robotics moving in dark places just like rats do it deliberately in dark- whisking mode.
Air object detection	**Ensure the safety of drones during flight**: Without the need for direct contact with the main body, whiskers can detect the variation in air pressure around the flying objects. [82]
Unmanned underwater vehicles	**Detect the water flow**: Whisker sensor is able to detect minute disturbances underwater [1,53], and reduce vortex induced vibrations [83].
Minimal invasive surgeries (MIS)	**Measure the mechanical behavior**: A micro whisker transducer is capable of detecting force, motion, and mechanical impedance from the electrical signals of the actuator itself [54,56,61].
Leak detection sensor	The authors are proposing a whisker that can be used to detect leakage in pipelines. By passing the whiskers through a piping system, the pressure change at the point of leak is high and hence the whisker can be sucked. These deformations indicate the presence of leak in that system (Figure 11).
Energy harvesting (micro-scale)	**Synthesized lead-free Na_0_._5_Bi_0_._5_TiO_3_ (NBT)**: the resulted whiskers had strip-like nanodomains which indicate the ferroelectric nature and exhibit 0.3% free strain at 9.5 kV/mm. The calculated value of field-induced S_max_/E_max_ reaches 300 pm/V [50,84].

**Figure 10:**
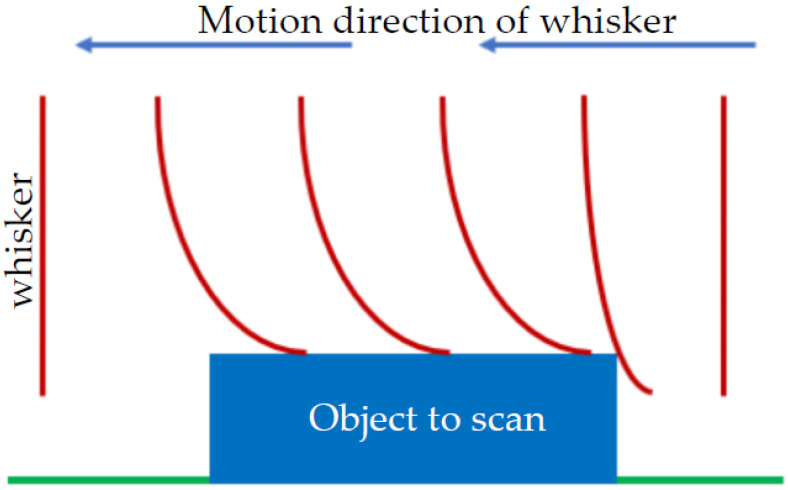
Schematic diagram of whisker sensors for studying surface textures.

**Figure 11:**
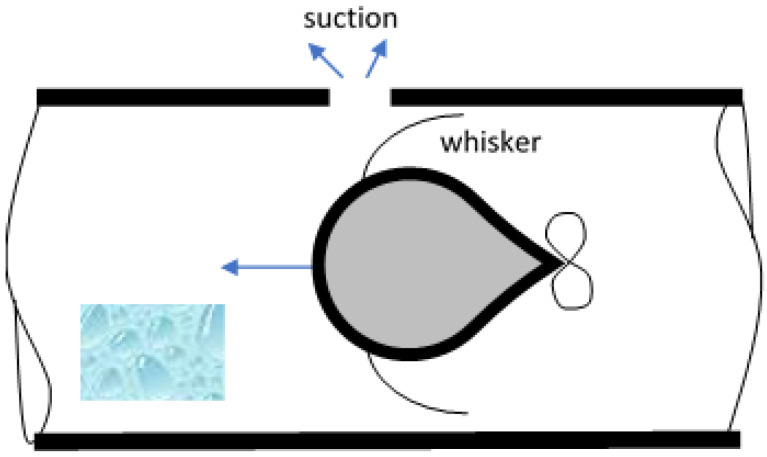
Leak detection sensor on water pipeline.

Whiskers can represent an absolute requirement for some applications and an alternative solution for others. They are needed in robots for leak detection (Figure 11) to allow the freedom of the robot in swimming away from the wall, whereby the whiskers can function in passive mode without affecting the robot. Whiskers may not be essential for profile inspection (Figure 10) but represent a potentially cheaper alternative solution, especially if they include surface roughness. Direct applications were adopted from rodent whiskers where biomimetic whiskers were designed by mimicking follicles. Measuring both the vertical and the horizontal deflection of a single whisker allows the detection of vertically shaped objects with a smooth surface. Two or more whiskers stacked vertically can recognize a vertical shape by observing the difference in their deflection amplitudes or the time shift of the deflection velocity peak. These results provide clues on how autonomous robots can improve their sensory capabilities with mechanical probes [84].

## 7. Research Gaps

Several research gaps were identified. As a future and alternative navigation system for autonomous robots, vibrissa sensing will take over in the development of whiskers moving diametrically in 360° steps to secure full perception of their surroundings. Long thin whiskers composed of conductive materials and/or with embedded sensors along the length can help in identifying proximity limits and fast decision making. Pulsed laser deposition can be used to create disulfide thin films, resulting in copper whiskers. It goes without saying that progress can be gained through innovations in materials science and engineering.

A whisker system is not only composed of whisker sensors that can sense their surroundings but also an inner network system analyzing data and developing perceptions. Strategies need to be developed for rapid object motion that rely on whiskers as external sensors. Artificial intelligence represents a key tool for further development in this regard.

Applications are needed for robots in extreme environments with respect to whiskers operating at high temperature and pressure. The main factor influencing the operation of whisker sensors is temperature. Wakabayashi et al. reported that increasing the temperature had a significant effect on resistance change, and the authors emphasized the need for further research [55]. Xie et al. also addressed this issue, stating that temperature increases bear a minor effect on performance [58].

In the work by Wu et al., the temperature effect was studied, but only up to 42 °C [29]. However, in the design tested by Harada et al., the temperature had no effect on the response, but the strain sensor was patterned on the surface of the whisker and was vulnerable to scratching [52]. As a result, more research into embedding strain gauges with an independent resistance to temperature changes is required with more active mode whisking.

Another possible area of study is the ability of tactile sensors to be integrated into various applications. For example, the use of e-whiskers to scan the topography of larger objects was highlighted by Mekid et al. [70]. The whiskers in this review were only used to scan small 2D objects; scanning larger topologies necessitates scaling up the size of these sensors, which may result in some complications. Whisker-based sensor technology should account for real-time measurements and feedback to ensure the ability to make quick decisions, especially in critical applications such as the use of whisker sensors in medical applications.

## 8. Conclusions

This paper presented a comprehensive literature review of the most recent whisker sensor designs inspired by mammalian whiskers without sensing along the length, but only at the base. This provides information about their surroundings and assists them in navigating difficult environments. As a result, great potential was shown in a variety of applications such as robotics, unmanned underwater vehicles (UUV), and minimally invasive surgery (MIS).

The design and performance of whiskers are heavily influenced by the materials used. Carbon nanoparticles, carbon nanotubes, and silver nanotubes are commonly used for sensing elements, while PDMS, PET, and PLA are commonly used for substrates. Furthermore, sensor materials must be strain-sensitive and robustly bonded to the substrate material to ensure long-term durability. As a result, the manufacturing technique becomes an important factor. In addition to screen printing and photolithography, common processes include 3D printing, molding, and spin-coating. Whiskers should be protected as well, which can be accomplished by embedding the sensitive material in the substrate, as shown in three designs in this review. Lastly, two potential research gaps were identified: investigations in extreme conditions, to determine the effect of temperature on sensitivity, and the possibility of scaling up the sensors to scan the topography of larger objects or even surfaces.

It is important to state that the sensing characteristics of the whisker depend on the concept chosen to emulate the sensing ability, the sensing materials, and the cross-section of the whisker. Furthermore, the system acquiring the data as well as analyzing and developing the intended function (e.g., perception) should also be considered.

## Data Availability

Not applicable.
